# Supplementary material for: Navigating Research Challenges: Collaborative Insights from a Research Retreat During a Healthcare Emergency in Puerto Rico
Source: Int J Environ Res Public Health. 2025 Apr 16;22(4):623. doi: 10.3390/ijerph22040623 (PMC12027351; doi:10.3390/ijerph22040623)
Supplement: Supplementary file 1 [file ijerph-22-00623-s001.zip › ijerph-3488746-supplementary materials final.pdf]

Navigating Research Challenges: Collaborative Insights from a Research Retreat During the COVID-19 Pandemic in Puerto Rico

Codebook – Dedoose Export

1. Challenges Category
   - 1.1 Remote Work
   - 1.2 Recruitment Limitations
   - 1.3 Incentive Distribution
   - 1.4 Protocols Revision
   - 1.5 Limited Personnel
   - 1.6 Low Lab Supplies
   - 1.7 Protecting Equipment
   - 1.8 Mental Health

2. Actions Taken Category
   - 2.1 Modifying Protocols
   - 2.2 Identifying Recruitment Strategies for Difficult Times
   - 2.3 Understanding Community Needs
   - 2.4 Utilizing Digital Platforms and Technologies to Connect with Research Staff and Participants
   - 2.5 Implementing Secure Strategies for Distributing Participant Incentives
   - 2.6 Redefining Priorities and Tasks
   - 2.7 Remote Work Implementation
   - 2.8 Personal Equipment Protection
   - 2.9 Online Purchasing

3. Emerged Opportunities Category
   - 3.1 New Supplement Applications
   - 3.2 New Protocols
   - 3.3 New Funding Opportunities
   - 3.4 Increased Focus on Publications
   - 3.5 Mental Health Tools to Support Personnel
   - 3.6 Gaining Knowledge of Technology Tools and Social Media Platforms
   - 3.7 Expanded Reach to Participants
   - 3.8 New Incentive Distribution System

Challenges	Actions Taken	Opportunities

The purpose of this survey is to evaluate the Activity *Navigating Unforeseen Challenges.... Lessons for Tomorrow* that was held on December 12, 2023, for the RCMI leaders and Investigators.

1. Indicate how much you agree or disagree with the following statements related to the activity.

	Completely Agree	Agree	Disagree	Completely Disagree	N/A
The coordination of this activity was handled effectively	◯	◯	◯	◯	◯
The allocated time for this activity was adequate.	◯	◯	◯	◯	◯
The agenda for the activity was appropriate	◯	◯	◯	◯	◯
The place where the activity was held was appropriate.	◯	◯	◯	◯	◯
The activity contributed to my professional development.	◯	◯	◯	◯	◯
I would participate again in activities like these	◯	◯	◯	◯	◯

2. How do you rate the following presentations:

	Excellent	Good	Fair	Poor
COVID-19 Vaccine Uptake Study (PR-COVACUPS)	◯	◯	◯	◯
The Impact of COVID-19 on Maternal Health in Puerto Rico	◯	◯	◯	◯
Communicating Science to Lay Audiences	◯	◯	◯	◯

3. How do you rate the group activity: COVID-19 Challenges and Opportunities

◯ Excellent

◯ Good

◯ Fair

◯ Poor

4. How do you rate the activity overall

★ ★ ★ ★ ★

5. Comments and suggestions
